# Paving the Road for Chimeric Antigen Receptor T Cells: American Society for Transplantation and Cellular Therapy 80/20 Task Force Consensus on Challenges and Solutions to Improving Efficiency of Clinical Center Certification and Maintenance of Operations for Commercially Approved Immune Effector Cell Therapies

**DOI:** 10.1016/j.jtct.2023.01.021

**Published:** 2023-01-26

**Authors:** Sarah Nikiforow, Matthew J. Frigault, Noelle V. Frey, Rebecca A. Gardner, Krishna V. Komanduri, Miguel-Angel Perales, Partow Kebriaei, Phyllis Irene Warkentin, Marcelo Pasquini, Joy Lynn Aho, Bruce L. Levine, Helen E. Heslop, Tracey L. Hlucky, Karen Habucky, Mecide Gharibo, Madan Jagasia, Frederick L. Locke

**Affiliations:** 1 Hematologic Malignancies, Medical Oncology, Dana-Farber Cancer Institute, Boston, Massachusetts; 2 Hematopoietic Cell Transplant and Cell Therapy Program, Massachusetts General Hospital, Boston, Massachusetts; 3 Medicine, Hematology Oncology, University of Pennsylvania, Abramson Cancer Center, Philadelphia, Pennsylvania; 4 Dept Of Pediatrics, Division of Hematology/Oncology, Seattle Children’s/University of Washington, Seattle, Washington; 5 Helen Diller Family Comprehensive Cancer Center and Division of Hematology and Oncology, Department of Medicine, University of California, San Francisco, San Francisco, California; 6 Department of Medicine, Adult Bone Marrow Transplantation Service, Memorial Sloan Kettering Cancer Center, New York, New York; 7 Stem Cell Transplantation and Cellular Therapy, MD Anderson Cancer Center, Houston, Texas; 8 Pathology/Microbiology, University of Nebraska Medical Center and Foundation for the Accreditation of Cellular Therapy, Omaha, Nebraska; 9 Medicine, Hematology/Oncology, Center for International Blood & Marrow Transplant Research, Milwaukee, Wisconsin; 10 Product and Innovation, Provider Services, National Marrow Donor Program/Be The Match, Minneapolis, Minnesota; 11 Pathology and Laboratory Medicine, Center for Cellular Immunotherapies, University of Pennsylvania, Philadelphia, Pennsylvania; 12 Center for Cell and Gene Therapy, Baylor College of Medicine, Houston, Texas; 13 Product Distribution Quality, Site Qualification, Kite Pharma/Gilead, Columbus, Ohio; 14 US Oncology Medical, Cell & Gene, Novartis Pharmaceutical Corporation, East Hanover, New Jersey; 15 US Medical Affairs, Hematology, Bristol Myers Squibb, Summit, New Jersey; 16 Medical Affairs, Iovance Biotherapeutics, San Carlos, California; 17 Blood and Marrow Transplant and Cellular Immunotherapy, Moffitt Cancer Center, Tampa, Florida

**Keywords:** CAR T cell therapy, Immune effector cell therapy, Standard of care, Treatment center, REMS

## Abstract

As the number and type of regulatory authority-approved cellular therapies grow, clinical treatment centers face a heavy burden of duplicative documentation around initial qualification, ongoing auditing, and reporting, with overlapping requirements from each manufacturer to ensure safe use of their specific product, which in the United States are stipulated under individual Food and Drug Administration (FDA) Biologic License Applications. The American Society for Transplantation and Cellular Therapy (ASTCT) convened the 80/20 Task Force to consider challenges and potential solutions to these issues. The Task Force proposed that 80% of manufacturers’ requirements for onboarding and ongoing operations of commercially available products could be standardized and streamlined. Task Force members interviewed dozens of stakeholders, including clinicians at large academic medical centers already using commercial and investigational immune effector cell (IEC) products, regulators, members of accrediting bodies and professional cellular therapy societies, and manufacturers of IEC therapies for oncologic indications. In November 2021, the Task Force organized and led virtual discussions in a public forum and at a private ASTCT 80/20 Workshop at the online AcCELLerate Forum, a cellular-therapy stakeholders’ meeting organized by the ASTCT, National Marrow Donor Program (NMDP), and Center for International Blood and Marrow Transplant Research (CIBMTR). At the workshop, approximately 60 stakeholders worked to identify and prioritize common challenges in onboarding and maintenance of operations at clinical sites for commercial FDA-approved and future IEC therapies and ways to streamline the process. It was agreed that standardization would improve efficiency of onboarding, allowing more cost-effective, sustainable growth of approved IEC therapies at treatment centers, and facilitate wider access while maintaining safety and clinical success. This early but extensive survey of stakeholders resulted in 5 overarching suggestions for both established and emerging treatment centers: (1) eliminate duplication in accreditation and auditing of clinical sites; (2) define expectations for the education about and management of CAR-T therapy toxicities to potentially replace product-specific REMS programs; (3) streamline current REMS education, testing, and data reporting; (4) standardize information technology (IT) platforms supporting enrollment, clinical site-manufacturer communication, and logistics of maintaining chain of identity/chain of custody across multiple transportation steps; and (5) encourage the use of universal nomenclature by cell therapy manufacturers. Future discussions need to engage a broader range of stakeholders, including administrators, pharmacists, nurses, data coordinators, surgeons, pathologists, and those developing promising cellular therapies for solid tumors, as well as teams from smaller academic or community cancer center settings. Continued collaboration with stakeholders outside of clinical sites will include accrediting bodies/auditors, established and emerging cell therapy companies, software developers, professional societies, and the patients who receive these therapies. Active dialog with government regulators remains essential. Such joint efforts are critical as the number of IEC therapies for myriad oncologic and nononcologic indications grows.

## INTRODUCTION

### Background

As new commercial immune effector cell (IEC) therapies have been approved by the US Food and Drug Administration (FDA) and other national regulatory bodies, clinical treatment centers have faced a heavy burden of duplicative activities and documentation related to initial qualification, ongoing auditing, and reporting. Manufacturers’ requirements often overlap with one another. There is extensive redundancy with existing cellular therapy accreditation requirements and entities that for decades have audited apheresis and cell-manufacturing facilities and academic cell therapy programs. Currently, most clinicians who prescribe FDA-approved IEC therapies do so within, or use resources stemming from, hematopoietic cell transplantation (HCT) programs at academic medical centers [1]. All IEC therapies approved to date are for hematologic malignancies, but manufacturers of solid-tumor therapies are engaged in the license application process [2–5]. As the field grows, smaller centers and community oncology clinics are becoming interested in offering IEC therapies; however, any center, even one with existing cellular therapy resources, can be overwhelmed by various manufacturers’ requirements for initial accreditation, ongoing audits, provider education, outcomes reporting, product logistics, and more.

As of this writing, there are 6 genetically modified FDA-approved chimeric antigen receptor T cell (CAR-T) therapies [6–12], manufactured by 4 different companies [13,14]. The number and types of IEC therapies are exploding, including additional CAR-T therapies and T cells with engineered T cell receptors (TCR), tumor-infiltrating lymphocytes (TILs), virus-specific T cells, and genetically engineered or ex vivo-expanded natural killer cells. According to a recent report, 1200 such IEC therapies may be investigated in the next few years for oncologic and nononcologic indications [14]. This expansion represents a potential boon to patients, clinicians, and investigators but challenges the sustainability and scalability of the field if processes for initiating and maintaining safe use of approved products at clinical sites are not streamlined [15–17].

The historical evolution of the clinically and logistically complex HCT field may provide guidance for the IEC and broader cell therapy fields. The use of “minimally manipulated” peripheral blood-derived stem cell or mononuclear cell products and bone marrow-directed products in standard-of-care autologous and allogeneic HCT settings has remained outside of traditional FDA-approval pathways. A network of HCT clinicians and professional societies, as well as the US federal government, addressed 3 areas of concern as the HCT field developed: (1) quality of procurement, processing, and administration of a cellular product with safe clinical management of the recipient; (2) aggregating data to assess safety and effectiveness within institutions and across the field, an effort supported by the federal government and embraced by insurance providers with publicly available clinical center HCT performance data; and (3) standardization of labeling and shipping logistics to ensure chain of identify/chain of custody as cellular products started to traverse the globe.

The following resources, among others, emerged:

1. Voluntary participation by clinical sites in accreditation of their quality programs for apheresis or marrow harvest sites (eg, Association for the Advancement of Blood & Biotherapies [AABB] [18], the NMDP [19], Foundation for Accreditation of Cellular Therapy [FACT] [20]), processing facilities (eg, FACT), and entire clinical programs (eg, FACT). The ASTCT [21], formerly ASBMT and International Society for Cell & Gene Therapy (ISCT) [22] jointly formed FACT in 1996; approximately 90% of eligible allogeneic HCT programs in the US today are FACT-accredited (167 accredited). FACT accreditation is a requirement for most major payers to qualify as a Center of Excellence within their networks. In partnership with the Joint Accreditation Committee ISCT-Europe & European Society for Blood and Marrow Transplantation (EBMT) (JACIE) [23], FACT continually revises HCT and “common” standards for non-HCT cellular therapies and accredits cellular therapy programs outside the United States.
2. Central repositories for clinical and product data, such as the CIBMTR [24,25], can be audited and shared for academic research purposes, quality initiatives, and clinical site performance review. The CIBMTR, administered jointly by the NMDP and Medical College of Wisconsin, collects data from more than 30 countries and is contracted by the US government to maintain the Stem Cell Therapeutics Outcomes Database (SCTOD) [25]. The SCTOD covers data submission for all allogeneic HCTs performed in the United States, as mandated by the C.W. Young Congressional Stem Cell Act, and facilitates publication of clinical center risk-adjusted outcomes [26] also used by payers to determine Center of Excellence status.
3. Standardized nomenclature, coding, labeling and ordering systems for cellular therapy products grew out of efforts of the International Council for Commonality in Blood Banking Automation (ICCBBA), Standards Coordinating Body (SCB) [27], NMDP [19], Parenteral Drug Association [28], and others, for example, Standard Terminology for Medicinal Products of Human Origin [29] and the Information Standard for Blood and Transplant (ISBT) 128 standard for labeling of medical products of human origin, which is used in 80 countries [30].

In contrast to HCT, genetically modified or ex vivo-expanded IEC cells, including CAR-T cells, are emerging as FDA-approved standard-of-care therapies following the route of a Biologic License Application (BLA). This pathway puts the onus for oversight of a quality program covering collection and manufacturing facilities, clinical site, and patient-care workflows on the manufacturer/sponsor. Critically, CAR-T therapies can cause unique, serious toxicities, both immediate or delayed, such as cytokine release syndrome (CRS), immune cell-associated neurotoxicity syndrome (ICANS), prolonged cytopenias, and a hemophagocytic lymphohistiocytosis-like syndrome (termed IEC-HS, for IEC-associated hemophagocytic syndrome, based on a recent ASTCT consensus project).

Each currently commercially available CAR-T product has been approved by the FDA with a product-specific Risk Evaluation and Mitigation Strategy (REMS) program to ensure safe use of these products by healthcare providers and patients. REMS programs require that clinicians and treatment center staff undergo specialized training and testing on toxicity management and report safety data to the manufacturer and the FDA, and that the treatment centers demonstrate the ability to provide medical treatment for potential toxicities (eg, tocilizumab) prior to CAR T administration. It is the manufacturer’s responsibility to ensure that its REMS program is implemented and followed. Although REMS programs for CAR-T products have the same core elements, REMS educational materials, toxicity management recommendations, and test questions and responses differ among products. REMS program initiation and monitoring generates additional onboarding, ongoing auditing, and reporting entities and requirements for sites delivering commercial IEC products, whereas the contribution to actual patient safety when layered over institutional quality and educational programs at clinical sites has been called into question. Even at high-volume IEC centers, concern is mounting regarding the resources needed to comply with existing auditing agencies, the varying manufacturer expectations, multiple REMS programs, and unique logistical workflows and portals for each product. Diverse stakeholders interviewed as part of this effort have expressed serious concerns that these burgeoning requirements eventually will limit patients’ access to potentially curative IEC therapies.

### Objectives

**Figure 1.**
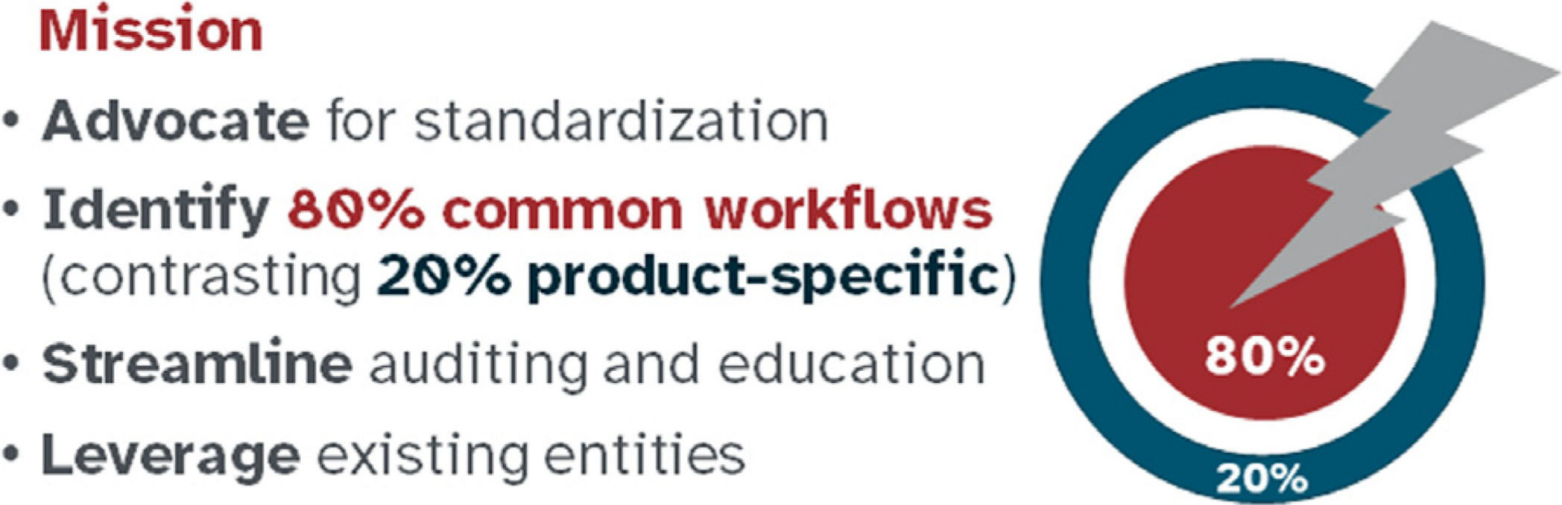
ASTCT 80/20 Task Force mission.

The ASTCT convened the 80/20 Taskforce based on the assumption that 80% of the requirements for clinical site onboarding and maintenance operations are similar enough among manufacturers of commercial IEC therapies and various accrediting bodies that they could be standardized and streamlined (Figure 1). Members of the 80/20 Task Force interviewed a broad spectrum of stakeholders and organized an online public forum and private workshop to identify the main areas of redundancy (ie, the proposed 80% common aspects of commercial IEC therapy clinical onboarding and ongoing operations between products and manufacturers) and opportunities for streamlining and standardization. A major focus was to identify how individual manufacturers might align on their expectations/requirements for clinical sites and whether HCT-derived resources could be used to satisfy/reduce duplication with industry requirements under their FDA BLAs.

The Task Force had the following objectives:

- Identify workflows common to current commercial IEC products and those close to completion of their development (the 80%) in 4 domains: (1) apheresis/cell procurement, cell processing/handling, and clinical site qualification and onboarding requirements; (2) ongoing auditing of clinical operations and data reporting; (3) educational and quality programs to ensure patient safety, including REMS compliance; and (4) IT and logistical interfaces.
- Highlight people, entities, and existing or newly required pathways that may streamline these processes to facilitate the sustainability and scalability of expected growth of IEC therapies.

## METHODS

We used a 3-pronged approach to gather insights from clinicians, regulators, accreditors, professional societies, and manufacturers of cell therapies (Table 1). First, in 2021, 2 authors (F.L. and S.N.) conducted interviews with representatives of professional societies and 5 commercial IEC manufacturing stakeholders with FDA-approved products or plans for imminent filing. Academic clinician perspectives were provided by ASTCT 80/20 Task Force members.

Second, a subset of these clinical and industry stakeholders discussed their perspectives with approximately 400 attendees at the online AcCELLerate Forum: Creating a Sustainable Ecosystem of Cell and Gene Therapy. This virtual forum, conducted on November 18–19, 2021, was sponsored by the ASTCT, CIBMTR, and NMDP.

Third, the 80/20 Task Force invited approximately 60 diverse stakeholders to attend a 2.5-hour ASTCT 80/20 Workshop, also held on November 18–19, 2021. To encourage candid discussion, the workshop was conducted under a verbal agreement that information and views shared by individual participants would not be taken as an official view of the entity that they were invited to represent. This workshop enabled staff at clinical sites (clinicians, administrators, and others) to freely engage in discussion with stakeholders nominated by relevant professional societies and commercial entities.

This process of obtaining consensus did not include interventions on either humans or animals and thus did not require approval by an institutional review board.

### Recommendations

**Figure 2.**
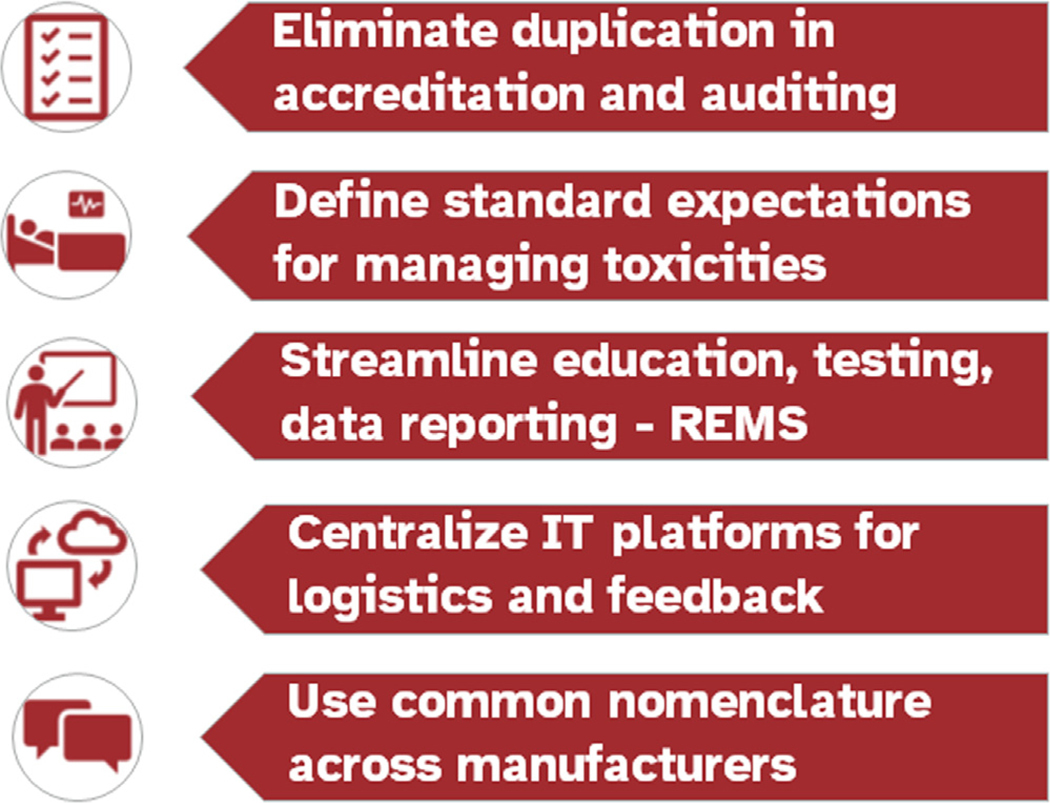
Potential solutions to challenges.

Stakeholders identified 5 recommendations that address overarching challenges associated with inefficiencies for clinical sites and commercial manufacturers (Figure 2). The voiced consensus was that patient safety and ongoing access to commercial IEC therapies could be improved if the community at large prioritized and addressed these issues [31].

### Recommendation 1. Eliminate duplication in accreditation and auditing of clinical sites

Initial accreditation/site evaluations and ongoing audits/monitoring visits currently occur on several levels at a clinical IEC site. The scope of evaluation can vary from the entire clinical program’s quality infrastructure to compliance of apheresis facility procedures with labeling, chain of custody, and chain of identify requirements. They can serve different purposes: FACT accreditation of an entire IEC or HCT program to enable coverage of cell therapy care by a payor (eg, California Medicaid, State of Massachusetts) versus annual compliance with REMS program requirements for a specific CAR-T product. Furthermore, the accrediting/auditing body for each purpose may differ (eg, FACT, NMDP, commercial manufacturer). There was stakeholder consensus that the safety parameters evaluated repeatedly in both initial and ongoing evaluations demonstrate significant overlap between different entities and areas of scrutiny.

1.1. A high level of initial qualification involves the entire clinical operation, particularly constituent pieces of the safety and quality program, such as:

- Standard operating procedures (SOPs) for program staff organization and oversight
- SOPs and processes for patient identification and tracking
- SOPs for infusion of cell therapy product
- SOPs and educational material for management of toxicities
- Evidence of involvement of specialty clinicians like neurologists and intensive care unit staff
- Processes for collecting, reviewing, and responding to patient outcomes across the program
- Processes for conducting quality improvements and corrective action plans.

These broad programmatic evaluations have been conducted historically by accrediting bodies such as FACT and more recently, by each new commercial CAR manufacturer de novo.

1.2. Individual sites and staff that perform specific functions are also evaluated at initial accreditation/qualification, including but not limited to:

- Apheresis facility (or operating room, eg, for TILs and other therapies in which tumor tissue is required as an IEC source material)
- Clinical site cell processing facility that ships out initial cell material, receives and stores manufactured product, and prepares the final cell product for administration
- Nursing staff who oversee cell infusion and patient monitoring
- Physicians who manage toxicities
- Pharmacy staff who ensure availability of medications for toxicity management, (including administration of supporting medications, eg, tocilizumab).

These facilities are often, but not always, part of the same clinical entity; for example, an apheresis facility may be outside of the treating hospital. Slightly different but overlapping subgroups of these sites have been evaluated historically by a combination of FACT, NMDP, and/or AABB and more recently by each individual commercial CAR-T manufacturer. Each CAR-T manufacturer also may require “product-specific” training for apheresis and cell processing for nursing, pharmacy and MD staff regardless of prior experience with cell therapy processes in general and commercial CAR-Ts in particular.

1.3. Finally, there are reaccreditation and ongoing audit/monitoring schedules—for example, FACT programmatic reaccreditation every 3 years, NMDP audits every 2 years, and product-specific REMS audits by each individual manufacturer at 6 and 12 months after infusion of the first product at a site and annually after that. Some commercial manufacturers also perform quality audits of apheresis and clinical site handling facilities every 2 to 3 years as well, consisting of SOP, training documentation, and lab practices review. For larger academic centers, these visits occur in addition to similar initial qualifications and ongoing monitoring visits for IEC research protocols, often by the same sponsor and for the same product that is commercially approved but being used in an investigational setting.

1.4. The ASTCT 80/20 Task Force proposes that a patchwork of resources covering much of this training and oversight is already available at the institutional and accrediting body level. The specific resources listed below are being increasingly used by specific manufacturers to satisfy such evaluations, which may obviate unique qualifications/audits by each individual manufacturer (Table 2).

- FACT: As of August 2022, 116 clinical programs were FACT-accredited under the Standards for Immune Effector Cells. Kite, a Gilead Company, the manufacturer of axicabtagene ciloleucel and brexucabtagene autoleucel, shared at the AcCELLerate Forum that it now accepts accreditation from FACT as satisfying most of Kite’s requirements for quality management systems at the clinical site, leading to an “abbreviated” audit, which requires review of less than one-half of the usual SOPs and cuts the time required by the clinical site by up to 75%. As of November 2021, 16 hospitals with FACT-accredited IEC programs had been qualified to dispense commercial axicabtagene ciloleucel and brexucabtagene autoleucel under this abbreviated approach. The second edition of FACT’s *Immune Effector Cells Standards* is currently under revision with manufacturers’ input solicited during the public comment period.
- NMDP: The NMDP created an approach to apheresis center evaluation using the Quality System Audit Program (QSAP) [32], plus an option to tailor an audit to cover common quality system elements on behalf of a manufacturer, thereby eliminating the need for the manufacturer to visit onsite. In several instances to date, such audit results have been shared with additional manufacturers to satisfy their audit needs as well.
- AABB: AABB [18] accreditation of apheresis centers provides another option for manufacturers to consider for field-recognized quality assessment.

The foregoing resources historically have not covered procurement of immune cells from sites other than peripheral blood apheresis centers and bone marrow harvesting suites, specifically tumor resections in operating rooms. The use of new starting materials for IEC therapies creates both an opportunity for and a challenge to existing auditing/accrediting entities in meeting the needs of relevant cell therapy manufacturers.

A risk-adapted or tiered algorithm for manufacturers to ensure that a site is adequately prepared to treat patients using their cell therapy product is preferred, according to the aforementioned Kite model. For example, if a center or individual site entity were FACT-accredited, then a minimal onsite or remote audit could be performed; if no program or site accreditation were present, then the audit by a manufacturer would be robust and lengthy. Potentially, wholesale ceding of initial accreditation and ongoing audits to third parties could be considered; for FACT-accredited sites, any remaining quality systems/site audits could be satisfied by a common audit, similar to the NMDP’s model for apheresis and cell therapy lab facilities, and then shared with multiple manufacturers.

Ideally, a small number of entities could accredit and audit all aspects of commercial cellular therapy delivery at a clinical site. This would require recognition that these audits satisfy most, if not all, manufacturers’ internal quality requirements for oversight under their FDA BLAs and/or REMS and quality expectations of the IEC field as a whole. Such entities also might help new centers launch by creating toolkits and education programs for staff based directly on their audit requirements. Although larger academic centers have this unwieldy process mapped out, at least for the 6 commercial CAR-T therapies already onboarded, onboarding may be a prohibitively daunting task for treatment locations with more limited IEC resources.

1.5. A flexible and modular model for accreditation. Although it is currently advantageous to centers with established HCT programs for commercial manufacturers to rely on professional society accreditation of their entire program (as noted above), smaller treatment centers are likely to have a paucity of experience and infrastructure related to cell therapies or may intend to offer a limited range of IEC therapies. Therefore, “quality management” programs and required oversight may be focused in scope, which will require determining a path to accreditation for administering one type of therapy without the need for the extensive documentation, reporting, and infrastructure requirements for full IEC clinical program accreditation.

For example, emerging cellular therapies for solid tumors, such as TILs, will involve solid tumor medical oncologists and specialists, such as surgeons and pathologists—different clinicians than have been involved in commercial IEC delivery to date. Operating rooms are already under extensive oversight (eg, Joint Commission) to ensure patient safety and chain of custody/identity of pathology specimens, and thus additional, duplicative audits by manufacturers or cell therapy accrediting bodies may not be relevant. Surgeons involved in tumor biopsy and TIL procurement are unlikely to benefit from the full 10 hours of cell therapy education yearly as required for clinicians administering cell therapies in FACT-accredited HCT or IEC clinical programs.

An option for a third party to provide a limited accreditation and audit schedule targeted to the services provided and meet the needs of specific manufacturers was strongly supported by the ASTCT 80/20 Task Force, with FACT actively investigating and recruiting expertise to address this proposal. The need for education to publicize awareness of these evolving options, especially for staff and administrators at sites new to the field, was emphasized.

Shifting to more focused modules for oversight mechanisms might enable manufacturers to outsource development and execution of these labor-intensive accreditation and auditing pieces. It also would allow treatment centers and the auditing body to avoid redundancy by leveraging portions of the process already completed for prior products. As more cellular therapy products are commercially developed to treat diverse conditions, the need for streamlined oversight will increase.

1.6. Hub-and-spoke model of accreditation. An alternative, complementary model might be for smaller treatment centers to partner with existing IEC centers and thus share knowledge and the burden of accreditation, onboarding, and audits. In this situation, a large center could act as regional hub to provide expertise and quality oversight, while patient care and delivery of commercial IEC products could be provided safely and effectively closer to home. This may be most feasible within larger medical systems that already have affiliated regional sites but more cumbersome for a truly nonaffiliated center to initiate financially, legally, and bureaucratically.

### Recommendation 2. Define expectations for education and management of CAR-T therapy toxicities to potentially replace product-specific REMS programs

CAR-T therapy has unique and sometimes delayed toxicities, including CRS, ICANS, cytopenias, and IEC-HS. To address safety concerns around these serious toxicities, each commercial CAR-T product FDA-approved to date must be administered under a REMS program as implemented and audited by the CAR-T manufacturer. This currently means that each manufacturer disseminates specific REMS program education materials to prescribing, administering, and dispensing staff with a subset of staff also undergoing protocol-specific REMS testing to confirm their product-specific knowledge. Experienced centers have evolved their own amalgamated, internally vetted CAR-T toxicity management algorithms yet are still expected to educate and test staff about REMS-specified management strategies that vary among products. Clinical stakeholders, while supporting the idea of systematic training to support clinical knowledge and safe use of these products, expressed concern that at this point in development of CAR-T therapies, the overlapping and repetitive education under individual REMS programs does not benefit clinician expertise and may even lead to provider confusion. Conversely, many clinicians felt that REMS educational material provided by a manufacturer is, in isolation, insufficient to ensure safe administration of CAR-T therapy, and that additional, often largely overlapping, institutionally generated SOPs, training, and oversight are needed and sufficient for optimal patient safety. (Toxicity reporting under REMS program requirements is addressed in a later section.)

2.1. The 80/20 Task Force posits that expert, local, and/or accrediting body-associated treatment guidelines and oversight for the management of common IEC toxicities are readily available within the community, and that individual manufacturer education and testing has become extraneous. Treatment centers, professional societies, and manufacturers should continue to jointly define standards for safety practices, toxicity grading schema, and management algorithms in genetically modified commercial IEC therapies (eg, efforts led by the ASTCT and others) [33,34]. Standard-of-care expert or institutional guidelines developed for the entire class of CAR-T products reduce conflicting recommendations for managing toxicities associated with commercial CAR-T products and typically are supplemented by specific caveats depending on the product or disease being treated. Similar to safety and quality practices designed by a given clinical HCT program and then routinely audited internally and by FACT (eg, SOPs for graft-versus-host disease prevention and management, management of graft failure, guidelines for prophylaxis and treatment of infections), internal processes already have been defined at centers currently administering commercial CAR-T products and represented at the Workshop. Workflows and SOPs cover recognizing risks for severe toxicity, algorithms to manage toxicity, required educational material for providers, and mechanisms to ensure sufficient stock of medications for treating toxicities. Furthermore, there already is an accrediting mechanism (eg, FACT) to ensure that such SOPs are in place at any IEC-accredited clinical center. Sharing of SOP templates with centers new to commercial IEC cell therapy by accrediting agencies could facilitate program development and accreditation.

2.2. Designing a future without REMS. Once the clinical community, in collaboration with manufacturers, establishes standard-of-care safety guidelines and ensures the necessary electronic educational and event-reporting platforms to support their implementation (see below), existing REMS programs may be phased out. Such guidelines should supersede existing risk mitigation measures and the current multiple and slightly different REMS-dictated recommendations for recognizing and managing toxicity of individual commercial CAR-T immunotherapies. The Task Force posits that safety guidelines established and maintained by the clinical cell therapy community should provide sufficient assurance for safe use of CAR-T therapies, as long as timely recognition of toxicities and management of adverse events are evaluated via initial implementation reviews and ongoing standard audits (eg, FACT). This possibility was discussed at the 2021 AcCELLerate Forum, at the 2021 80/20 Workshop, and, most recently, at the 2022 AcCELLerate Forum and December 2022 Cell Therapy Liaison Meeting with the FDA.

### Recommendation 3. Streamline current REMS education, testing, and data reporting

3.1. Focused training for specific staff. Currently, treatment centers need to train diverse staff to recognize and manage toxicities of cellular therapies. As stated above, each product has a relatively small amount of unique and specific educational requirements, yet current REMS training modules are both redundant in the basics of toxicity identification and are neither designed nor adequate to address critical details of cellular therapy administration for all roles involved (eg, physician, infusion nurse, apheresis staff, etc).

We suggest that REMS-specific training and any verification/testing could be modified to each person’s responsibilities. Many stakeholders at the Workshop felt that it is ultimately the treatment center’s responsibility to ensure adequate, relevant, and targeted training and certifications for specific staff. For example, it is not relevant for cell processing staff thawing a CAR-T product prior to infusion to be trained to answer questions pertaining to CRS grading and medical management in intensive care unit settings, as are physicians. Many centers initially administered such REMS testing for any staff member who prescribes, dispenses, or administers a commercial CAR-T product, which, depending on interpretation, can include technicians thawing CAR-T bags and bedside nurses performing i.v. infusions. Ideally, the educational content contained in any central training program should be both universal and flexible to accommodate new toxicities associated with emerging commercial IEC therapy products; however, each clinical institution should address which training subtleties need to be focused on specific staff.

3.2. Centralized online education and/or tracking of REMS testing. The ASTCT 80/20 Task Force recommends creation of a centralized, shared platform for general IEC education and testing of safety management strategies. Currently, centers must ensure that staff are trained on each individual product’s REMS program, which requires introduction of variable content into already complex clinical operations. This testing creates logistical challenges involving multiple electronic platforms across manufacturers, even potentially between products from the same manufacturer. A centralized platform would greatly reduce the administrative effort for REMS management at treatment centers. This may also lower costs and labor for manufacturers that currently create their educational material, perform training, and manage testing portals de novo. As shared at the AcCELLerate Forum by a representative from a CAR-T manufacturer, selected commercial stakeholders already have been working together with the Alliance for Regenerative Medicine to investigate a joint REMS program centered around consistent education, testing, and access.

An existing resource discussed among stakeholders was cell therapy educational modules created by the Society for Immunotherapy of Cancer (SITC) [35]. Specific SITC cell therapy modules addressing investigational immune-directed therapies, including TILs, have been launched recently. These do not exhaustively cover all staff subspecialities involved in a cell therapy program and are not CAR-T- or REMS-specific. However, similar models could be built upon or adapted as a unified effort to educate diverse staff on targeted aspects of cell therapy. Similarly, the ASTCT has a robust history of educational seminars designed to inform and train provider members and allied health professionals about different aspects of cell therapy, including CAR-T therapy. Such educational modules might yield certification or continuing medical education credit in place of REMS education that could be recognized as proof of training by quality programs at clinical sites, accreditation bodies, and manufacturers.

3.3. Reporting toxicity: Common data points and mechanisms. Beyond education, the REMS for each CAR-T product requires tracking of severe and unexpected toxicities following therapy. Although all current REMS programs mandate reporting of serious adverse events (SAEs), clinical sites have expressed difficulty interpreting which data points need to be reported and at what level of severity reporting is indicated. Currently, clinical centers do not report to a single entity, as they are expected to report to individual manufacturers or directly to FDA.

One current CAR-T REMS program indicates clinical sites must report any SAE suggestive of CRS or neurologic toxicities to either the manufacturer by phone, email, or website or to the FDA by phone or MedWatch form [36]. SAEs are defined as any adverse experience occurring at any dose that results in any of the following outcomes: death, a life-threatening adverse experience, inpatient hospitalization or prolongation of existing hospitalization, a persistent or significant disability/incapacity, or a congenital anomaly/birth defect. Each REMS stipulates the expectation that such events will be reported if observed following CAR T; however, center representatives expressed uncertainty regarding the reporting of events that are very unlikely or entirely unattributable to CAR-T therapy and confusion about the degree of longitudinal oversight treating centers should provide. Therefore, each institution delivering commercial CAR-T products has determined its own guidelines for what, when, and how to report as compliance with reporting “any” SAE.

Reporting to the FDA through MedWatch forms requires individual data entry of numerous fields, which involves significant labor. Each manufacturer has onerous interfaces and duplicative mechanisms/forms for reporting, which often generate extensive follow-up queries with questions that are duplicative with the initial submission and too onerous for clinical staff to complete. Therefore, many large academic centers now report patient, product, effectiveness, and toxicity data after receipt of commercial CAR-T therapy to a single entity, the CIBMTR, using their IEC therapy forms and data fields (see below), which then can return a report that could be shared with manufacturers to satisfy REMS reporting. Clinical sites now often only report directly to the manufacturer or the FDA on a very restricted subset of severe events (eg, deaths within 30 days of CAR-T therapy).

3.4. Centralized reporting of IEC outcomes. The Cellular Immunotherapy Data Resource (CIDR) of the CIBMTR was established with federal grant support to collect demographic, toxicity, and outcome information of patients treated with cell therapy. Although there is currently no federal mandate to collect data on IEC recipients as there is for allogeneic HCT [37], manufacturers of all 6 commercially approved CAR-T products have contracted with the CIBMTR to collect data to help meet their requirements for post-marketing safety studies. The CIBMTR has established mechanisms to send data back to the clinical sites (ie, can serve as the primary repository for an individual clinical program), which can then forward consistent agreed-upon data on a standard time frame to each manufacturer. Many clinical sites have utilized this workflow to cover toxicity reporting requirements and to spur ongoing research in the field through academic-sponsored and manufacturer-supported analyses, while reporting only the most concerning toxicities directly to the FDA or sponsor.

The CIBMTR’s standard data fields and clear reporting process are very familiar to allogeneic transplant centers, albeit different data fields have been created for IEC products in general, for commercial CAR-Ts in particular, and continue to evolve under the CIDR’s auspices to cover newer investigational cellular therapies. The CIBMTR’s process as currently used by clinical sites has clarified expectations regarding nomenclature and workflows for toxicity reporting and has greatly reduced the burden on treating centers. This data repository specifically for recipients of commercial CAR-T products also has supported publications by manufacturers and academic investigators in the field that robustly address real-world questions from more than 7500 recipients of commercial CAR-T products [25]. This CIDR/CIBMTR model remains attractive for postmarketing data of other commercial IEC therapies in the future and can continue to support large-scale observational studies to advance the field. To date, no payer reporting based on IEC outcomes to determine Center of Excellent status has been proposed, but this possibility has been discussed.

The ASTCT 80/20 Task Force strongly advocates a single, common platform or portal with standard terminology to report on safety and effectiveness. The FDA and commercial and clinical stakeholders will need to continue collaborations to reach consensus on what is expected/required for data acquisition across the commercial IEC field as it diversifies.

### Recommendation 4. Standardize IT platforms for enrollment, logistics of chain of identity/chain of custody, and communication

To date, each commercial IEC product manufacturer has invested in a different, proprietary computer application/portal or even multiple portals to facilitate patient enrollment, manufacturing dates, and shipping expectations. This was identified as a major area of concern by both clinical and manufacturing stakeholders. As more products are approved, the sheer number of unique IT platforms and variety of communication pathways will eventually overwhelm clinical site staff and can be extremely expensive for each manufacturer to support. This will limit both access to therapy at the site level and patient safety from all angles. Suggestions for improvement ranged from simple (eg, allow access to each portal via an institution’s single sign-on identifier and password for a given staff member) to more complex (eg, standardizing nomenclature and data points entered/communicated between programs/portals) to the ideal scenario of a single platform across all commercial IEC manufacturers.

To avoid errors, standardized modes and methods of communication are needed to transmit important logistical information about identity and location of the product over time and to synchronize IEC commercial manufacturing with other therapies and patient care. In the current state, booking of manufacturing slots and notification of the date that manufactured product will be delivered to the site varies across manufacturers; successful completion of each manufacturing step may be reflected in the IT platform, or only the final release may be communicated by phone call or email. Similarly, if a product is deemed out of specification or unacceptable for commercial use, processes for notification are wildly disparate between manufacturers and not always timely or complete. Many centers have identified communication fatigue. When manufacturers send repetitive emails addressed to many staff at the same institution, staff are uncertain about who is responsible for acting on the information and when action is necessary. Conversely, a portal may allow for notification of only 1 or 2 site representatives, which is insufficient for coordinated clinical action on the patient’s behalf. Ideally, bidirectional and similar feedback between sites and manufacturers could be provided in a templated, trackable, and sustainable manner through these portals.

4.1 IT platforms shared by multiple products and manufacturers. Multiple initiatives are underway to address these issues, including one workstream and IT platform championed by Deloitte’s Industry Working Group and a separate Accenture-sponsored software package. At the AcCELLerate Forum, an NMDP representative reviewed decades of experience with the universal ordering system Traxis that evolved to serve the global allogeneic HCT community and discussed how mirroring a similar overlay and support system for commercial IEC therapy logistics may be advantageous. Workshop participants voiced concern that proliferation of efforts to establish “new” IT platforms may not actually help with consolidation of efforts any more than individual manufacturers’ platforms. Consolidation within the field to 2 or 3 IT platforms may be most feasible.

### Recommendation 5. Use universal nomenclature as much as possible

To ensure trackable chain of custody/chain of identity, which is crucial to ensuring that the appropriate patient is infused with the correct therapeutic product, the ASTCT 80/20 Task Force highly recommends that commercial IEC manufacturers adopt a universal set of labels for initial leukaphereses and for IEC products (particularly CAR-T genetically modified products) on return to the clinical site. ISBT 128 labels reflect a standard for medical products of human origin accepted in 80 countries. A standard recently written by an SCB workgroup and endorsed by a subgroup of commercial IEC manufacturers enacts use of a standard ISBT 128 label for leukapheresis products upstream of commercial CAR-T manufacturing. The ICCBBA, through its Cellular Therapy Coding and Labeling Advisory Group, has made progress [38] toward a label template for clinical trial and commercial cellular products to facilitate chain of identity maintenance across all clinical and manufacturing sites, but this work is still underway. Even if not fully ISBT 128-compliant, a strong recommendation was made to incorporate the universally recognized and globally unique donor identification number (DIN) into labels to track products from initial collection to final bedside infusion, as opposed to a sponsor patient/product number, which actually might not be unique across manufacturers. Ideally from a clinical site perspective, labels would contain unique identifiers that also align with local medical records, for example, full patient name and institutional medical record number.

Preferably, all commercial IEC manufacturers also would use unified terminology and nomenclature in their IT portals and communications, instead of the wide variety of terms used now (eg, leukapheresis versus apheresis, delivery versus final product delivery, treatment versus therapy). Current ordering systems typically ask the treatment center to identify a unique name for the patient; however, the format for this varies (eg, first, middle, last names versus last, middle initial, first name, etc), and this might not be modifiable if an error or a change is discovered. The nomenclature for the product may be “Lot #,” “Batch #,” “ID #,” or “JOIN #,” or a combination instead of or in addition to the donor identification number. Use of standard, agreed-upon nomenclature for product ordering, chain of custody/chain of identity steps, and labels on products returned to the center will create a safer environment for patients and avoid accidents and errors.

### Discussion Points for the Future

In addition, several other points, briefly introduced below, are outside of our initial scope but merit further investigation and discussion.

#### Standardize processes in pivotal trials

Many logistical nuances between different IEC commercial therapies stem from what was practiced in the Investigational New Drug Application for pivotal/registration investigational trials and became locked in at the time of FDA approval of the commercial BLA. These include chain of custody and chain of identity details, leukapheresis volumes and plasma addition, ability to sample apheresis and manufactured products at the clinical site, composition of cryopreservation media and freezing approaches, procedures for couriers, and criteria for shipping containers and accompanying documentation. A standard approach to these steps also may aid research efforts, including identification of biomarkers of toxicity and efficacy [39]. Future topics of discussion include whether simple bridging studies could be designed to change labels or noncritical logistics linked to already approved IEC products to a common standard and whether any guidelines for commercial IEC products could be incorporated into future registration clinical trials to streamline eventual commercial transition.

#### Reimbursement/financial challenges

Administrative leaders from multiple clinical sites raised concerns about reimbursement paradigms for commercial CAR-T products and associated clinical care around administration. There is concern that some payers do not “adequately” reimburse for cellular therapies, with great disparities between payers. The frequent need to negotiate payer agreements for individual patients is onerous and can delay access to therapies. These detrimental impacts weigh most heavily on patient populations historically affected by barriers to access cancer therapies, including underrepresented minorities and socioeconomically disadvantaged individuals [40]. To ensure appropriate reimbursement, professional societies may advocate for more standardized payment structures and help educate centers on accurate coding and claims approaches.

Additionally, the need for commercial IEC clinical sites to meet multiple, redundant requirements for reporting and auditing has required hiring multiple new staff/roles and created a time and financial burden on clinical site personnel that is not reimbursable under current physician fee-for-service or hospital diagnosis-related group billing models. If a core institutional group could cover the streamlined 80% of accrediting/auditing visits and IT/logistical requirements common to and standardized among commercial IEC therapies, it then might be feasible to staff the more limited 20% requirements for individual products and manufacturers. If a given manufacturer chooses not to accept relevant third-party accreditation, then centers likely will move to request reimbursement for the excess time spent or choose a different manufacturer if more than one option is available for a given indication. Future meetings on financial sustainability of the general IEC commercial field and staffing at clinical sites will require active engagement from hospital executives and payers.

#### Continued engagement of the broader clinical cell and gene therapy field

The 80/20 Task Force and Workshop started with a defined scope by necessity, which focused on FDA-approved IEC products for cancer indications. However, recent commercial approval of the first 2 genetically modified stem cell products, advancement of engineered T cell receptors (TCR) and TIL manufacturers toward the commercial application process, and expansion of investigational cell therapy into different cell types such as mesenchymal stromal cells and induced pluripotent stem cells, raise the question of how our IEC-based initiative described above might have ramifications or provide a model for other cell therapies, clinicians, and manufacturers. We anticipate that our professional organizations, such as ASTCT, SITC, ISCT, ICBBAA, SCB, CIBMTR, FACT, NMDP, American Society of Gene & Cell Therapy, American Society of Clinical Oncology, American Society for Hematology, and others, will continue to collaborate, seek input from stakeholders, and advocate for streamlined paradigms to sustain growth across the cell therapy field.

Finally, our workshop was primarily US-based, but similar challenges have been identified by the European-based GoCART Coalition [41] sponsored by the EBMT. We look forward to international cooperation through the ASTCT, EBMT, and ISCT on these issues as regulatory environments outside of the United States present some similar and some unique scenarios for the expansion of CAR-T and IEC therapies across the global stage.

## CONCLUSION

The ASTCT 80/20 Task Force, through one-on-one stakeholder meetings, the public AcCELLerate Forum, and a dedicated Workshop with approximately 60 stakeholders from very diverse backgrounds, yielded consensus on key issues impacting safe and efficient commercial IEC delivery and agreement on several mutually beneficial approaches, some of which can leverage existing HCT-derived resources such as the NMDP, FACT, and CIBMTR. This early but extensive discussion resulted in 5 overarching suggestions: (1) eliminate duplication in accreditation and auditing of clinical sites; (2) define expectations for education and management of CAR-T cell therapy toxicities to potentially replace product-specific REMS programs; (3) streamline current REMS education, testing, and data reporting; (4) standardize IT platforms for enrollment, logistics of maintaining chain of identity/chain of custody across multiple transportation steps, and clinical site-manufacturer communication; and (5) promote use of universal nomenclature, as much as possible, by cell therapy manufacturers.

These recommendations were generated in the context of currently available and potential future commercial IEC therapies in cancer. We recognize that different IEC therapies currently under clinical investigation may reveal new challenges and require different solutions for safe and efficient delivery. IEC applications in solid tumors, beyond the current hematologic malignancy space, will entail different workflows. Commercial partners are exploring administration of commercial IEC cell therapy outside of the standard academic transplantation centers. Engagement with “emerging” IEC manufacturers/sponsor prior to commercial approval should allow the field to explore approaches to streamlining before rather than after FDA BLA approval. Momentum on our consensus suggestions and active engagement with these additional stakeholders has now transitioned to the 80/20 Subcommittee under the ASTCT’s Committee on Cellular Therapy, with ongoing presentations and workshops planned. These future efforts will include solid tumor clinicians, surgeons, pathologists, pharmacists, data coordinators, and hospital administrators, particularly at clinical sites new to delivering IEC therapies. We look forward to continued collaboration with the FDA, accrediting/auditing bodies, professional societies, the CIBMTR, software developers, and an ever-increasing array of industry partners. The common goal for all is sustainability and scalability of the IEC commercial field to allow patients safe access to the most effective therapies possible.

## Figures and Tables

**Table 1:** ASTCT 80/20 Task Force Activities and Stakeholder Engagement

Activities	Regulatory Body	Academic Institutions/Cancer Centers	Professional Societies	Commercial Entities
Step 1: Stakeholders initially interviewed by ASTCT 80/20 Task Force leadership	FDA	Moffitt Cancer Center Dana-Farber Cancer Institute MD Anderson Cancer Center Fred Hutchinson Cancer Center Massachusetts General Hospital University of Pennsylvania Memorial Sloan Kettering Cancer Center	CIBMTR FACT ISCT NMDP SITC	Bristol Myers Squibb Iovance Janssen Kite/Gilead Novartis
Step 2: Speakers or panel participants in 2021 AcCELLerate Forum [42]	FDA		ASTCT CIBMTR/CIDR FACT NMDP	Iovance Janssen Kite/Gilead
Step 3: 2021 80/20 Workshop participants (representing the following roles: administrative/financial, cell processing, nursing, physicians, quality)		Baylor College of Medicine Children’s National Cancer Center Dana-Farber Cancer Institute Emory University Fred Hutchinson Cancer Center MD Anderson Cancer Center Massachusetts General Hospital Mayo Cancer Center Memorial Sloan Kettering Cancer Center Moffitt Cancer Center Ohio State University University of Miami Sarah Cannon Cancer Center Stanford University University of Chicago University of Kansas University of North Carolina University of Nebraska University of Pennsylvania Washington University of St Louis	ASTCT CIBMTR/CIDR FACT ISCT NMDP	A2 Bio Accenture Roche Allogene Bristol Meyers Squibb Bluebird Bio Carsgen Deloitte Instil Bio Iovance Janssen Kite/Gilead Legend Biotech Miltenyi Novartis Precigen Tmunity Trimvira

Individuals from the listed entities participated in the indicated stages of development and discussion. During the Workshop, opinions expressed were considered those of the individuals and not necessarily representative of an affiliated academic center, professional society, or commercial employer.

**Table 2:** ASTCT 80/20 Task Force Stakeholder Recommendations for Immune Effector Cell Therapy Standardization

	ASTCT 80/20 Task Force and Stakeholder Goals	Strategies in Development	Potential Future Initiatives
1	Eliminate duplication in accreditation and auditing of clinical sites	• Risk-adapted or tiered algorithms to sponsor auditing, eg, using FACT accreditation • Existing accreditation entities with shared reports/findings, ie, FACT, NMDP, AABB	• Modularization of auditing for specific site or manufacturer needs • Hub-and-spoke model of quality programs/accreditation for smaller centers
2	Define standard and uniform safety guidelines for managing CAR-T cell therapy toxicities to potentially replace product-specific REMS programs	• Expert consensus guidelines exist on treatment management strategies, eg, NCCN	• Expert local and/or accrediting body-based treatment guidelines and oversight
3	Streamline education, testing and data reporting on CAR-T toxicities currently performed under REMS	• Commercial collaborations are considering a shared REMS program and/or centralized testing	• Centrally available education modules geared to individual roles within clinical sites • Agreement on common data points and central mechanism for reporting, ie, CIBMTR
4	Standardize IT platforms for enrollment, logistics of maintaining chain of identity/chain of custody across multiple transportation steps, and clinical site-manufacturer communication	• Limited number of portals using agreed-upon nomenclature, identifiers, and processes	• Limited number of portals using agreed-upon nomenclature, identifiers, and processes
5	Use of universal nomenclature, as much as possible, by cell therapy manufacturers	• ICCBBA/ISBT 128 labeling standards for apheresis and final manufactured products • Standards coordinating body initiatives	• Recognition of common workflows for apheresis collections, labels, and transportation documentation
